# Sickle cell disease: a case report of renal amyloidosis

**DOI:** 10.1186/s12882-018-1047-6

**Published:** 2018-10-10

**Authors:** Ann Bugeja, Paula Blanco, Edward G. Clark, Manish M. Sood

**Affiliations:** 1 Division of Nephrology, Department of Medicine, The Ottawa Hospital and University of Ottawa, Riverside Campus, 1967 Riverside Drive, Ottawa, ON K1H 7W9 Canada; 2 Department of Pathology, The Ottawa Hospital and University of Ottawa, General Campus, 501 Smyth Road, Ottawa, ON K1H 7W9 Canada

**Keywords:** Proteinuria, Sickle cell, Amyloidosis, Chronic kidney disease

## Abstract

**Background:**

The development of proteinuria and reduced glomerular filtration rate is associated with higher mortality among patients with sickle cell disease (SCD). AA amyloidosis, also associated with increased mortality, in SCD is rare. We present a case of a woman with homozygous sickle cell disease with nephrotic syndrome and antibodies to double stranded DNA without clinical features of systemic lupus erythematosus. Kidney biopsy reveals AA amyloidosis and is the first report of concomitant AA amyloidosis with antibodies to double stranded DNA in SCD.

**Case presentation:**

A 40-year-old Central African woman with homozygous sickle cell disease and history of vaso-occlusive pain crises undergoes kidney biopsy for nephrotic-range proteinuria. Kidney biopsy reveals AA type amyloidosis, which is a rare manifestation of SCD in the kidney. Her anemia worsens with an ACE inhibitor, initiated to reduce proteinuria and limit GFR decline, so it was discontinued. Hydroxyurea, shown to decrease the frequency of vaso-occlusive crises and lower proteinuria, was subsequently initiated but then discontinued due to worsening anemia. Unfortunately, her glomerular filtration rate worsens.

**Conclusions:**

AA amyloidosis and antibodies to double stranded DNA can occur in sickle cell disease. ACE inhibition and hydroxyurea decrease proteinuria so they may limit progression of chronic kidney disease. Hydroxyurea also decreases frequency of vaso-occlusive pain crises so it might be helpful in limiting progression of renal AA amyloidosis. However, further studies are needed to determine optimal treatment strategies for AA amyloidosis in sickle cell disease.

## Background

Sickle cell disease frequently affects kidney structure and function. Early glomerular hyperfiltration can lead to glomerular hypertrophy then proteinuria [1] with glomerulosclerosis and decreased glomerular filtration rate (GFR) in sickle cell disease [1]. Focal segmental glomerulosclerosis is the most frequent glomerulopathy seen in patients with sickle cell disease [1]. Membranoproliferative glomerulonephritis and thrombotic microangiopathy have also been described [1]. However, nephrotic-range proteinuria has been only noted in approximately 4% of patients with proteinuria and is associated with reduced GFR and increased mortality [1]. We present the first report of concomitant AA amyloidosis with antibodies to double stranded DNA in SCD.

## Case presentation

### Clinical history and initial laboratory data

A 40-year-old Central African woman with homozygous sickle cell disease was referred for evaluation of proteinuria. During the past 5 years, her serum creatinine (Scr) level ranged from 0.35 mg/dL – 0.70 mg/dL (corresponding to estimated GFR of 126–164 mL/min/1.73m^2^, using the corrected CKD-EPI [Chronic Kidney Disease Epidemiology Collaboration] equation for race. Five years prior to nephrology presentation, her urine albumin to creatinine ratio (ACR) was 610 mg/g; 2 months ago, it was 7779 mg/g.

At nephrology evaluation, she reported multiple vaso-occlusive pain crises as a child. She had a vaso-occlusive pain crisis and required red cell transfusion following a therapeutic abortion 5 years ago. Six emergency room visits followed for vaso-occlusive pain crises. She has proliferative sickle cell retinopathy and restrictive lung disease. Folic acid was her only medication and she took acetaminophen for a vaso-occlusive pain crisis 2 months ago.

Her physical examination revealed a non-obese woman with a blood pressure of 120/70 mmHg and heart rate 94 beats per minute and regular. She did not have any flow murmurs on precordial examination and she had bilateral ankle edema. The rest of her physical examination was normal. Laboratory investigations (Table 1) revealed: Scr 0.94 mg/dL, estimated GFR, 88 mL/min/1.73m^2^ and hemoglobin 64 g/L. Urinalysis showed 2+ blood, 3+ protein and urine microscopy revealed 5–30 red blood cells without casts. Urine ACR is 6089 mg/g. Serologic workup revealed an anti-nuclear antibody titer of 1:320 and anti-double stranded DNA titer 72 IU/ml by ELISA. Investigations for sarcoidosis were not performed. Abdominal ultrasonography revealed that the length of both kidneys was 12 cm. She consented to kidney biopsy under ultrasonography guidance to diagnose the cause of her nephrotic syndrome.

**Table 1:** Laboratory results at initial nephrology visit

Parameter	Value	Reference Range
Blood		
Creatinine (mg/dL)	0.90	0.25–0.85
eGFR (ml/min per 1.73 m^2^))	88	≥ 90
Sodium (mEq/L)	141	135–145
Potassium (mEq/L)	3.0	3.5–5.0
Chloride (mEq/L)	106	98–107
Corrected serum calcium (mg/dL)	10.0	8.5–10.1
Albumin (g/dl)	2.2	3.4–4.6
Hemoglobin (g/dl)	64	115–155
Mean corpuscular volume (fL)	69	80–100
Hematocrit (%)	20	38–50
Reticulocyte count (%)	38	0.5–1.5
Lactate dehydrogenase (U/L)	258	100–205
Glucose, fasting (mg/dl)	95	72–198
Cholesterol, total (mg/dl)	213	135–201
LDL (mg/dl)	135	135–193
Triglycerides (mg/dl)	163	≤ 177
HDL (mg/dl)	46	≥ 50
C3 (mg/dl)	142	90–180
C4 (mg/dl)	20	10–40
Hepatitis B surface antigen	negative	negative
Hepatitis C surface antibody	negative	negative
HIV	negative	negative
Anti-nuclear antibody	1:320	negative
Anti-double stranded DNA (IU/ml)	72	< 30
Serum monoclonal protein	Negative	negative
Urine		
Red blood cell (/HPF)	5–30	0–2
Albumin/creatinine ratio (mg/g)	6089	≤ 18
Monoclonal protein	Negative	negative

*eGFR* estimated glomerular filtration rate by CKD-epi equation, *LDL* low density lipoprotein, *HDL* high-density lipoprotein, *HIV* human immunodeficiency virus, *HPF* high power field

### Kidney biopsy results

**Fig. 1:**
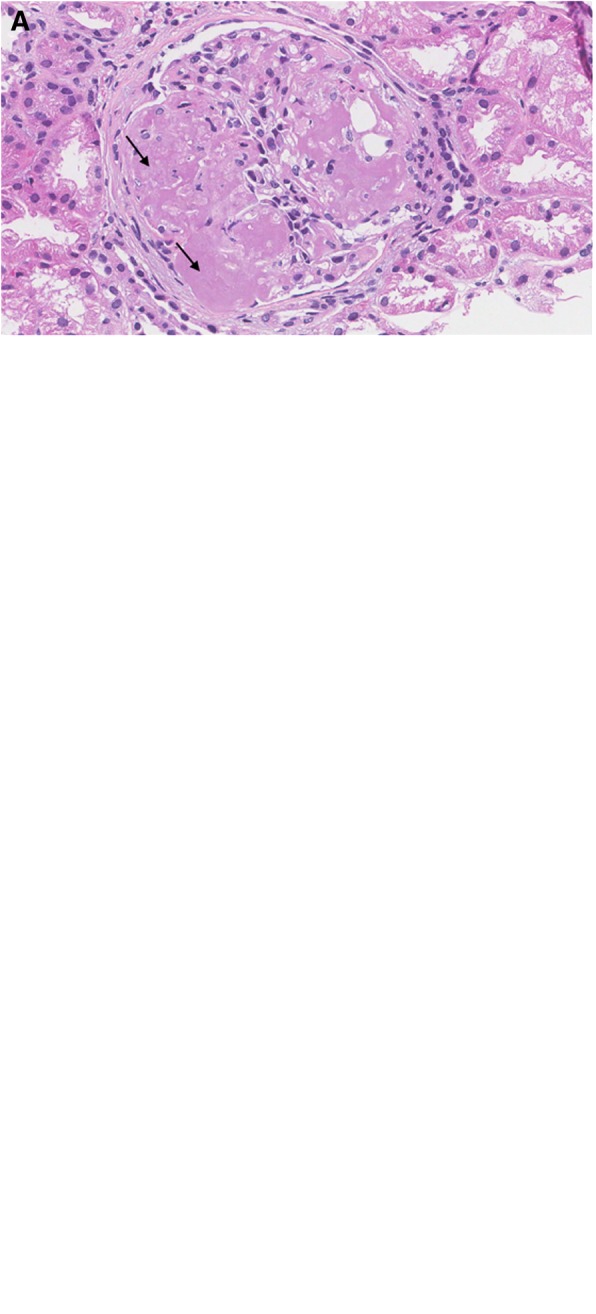
Kidney biopsy specimen. **a** Hematoxylin and eosin stain (original magnification x 200) with arrows pointing to nodular material consistent with amyloid

The kidney biopsy specimen contained up to 15 glomeruli, 3 of which were globally sclerosed. There was no significant glomerular hypertrophy. Most glomeruli showed prominent nodular accumulation of amorphous, eosinophilic, weakly PAS positive, non-argyrophilic (staining properties of amyloid) material (Fig. 1a, arrow). Congo red stain confirmed that this material exhibited the characteristic apple green birefringence of amyloid when examined under polarized light. Amyloid was type AA as demonstrated by immunohistochemistry. Amyloid extensively extended into arterioles (Fig. 1b, arrow). There was patchy but significant deposition of amyloid in the walls of tubules with associated epithelial injury. Prussian blue stain for iron, often seen in tubular epithelial cells in sickle cell nephropathy, was negative. There was mild interstitial fibrosis and tubular atrophy. Arteries showed wall thickening by amyloid as well as multilayering of the internal elastic lamina. Immunofluorescence showed weak granular non-specific glomerular staining for IgM and C3. There was no staining for IgG, IgA, or κ and λ light chains. Electron microscopy showed deposition of non-branching randomly arranged fibrils, consistent with amyloid, predominantly in mesangial areas but also along and permeating the glomerular basement membrane. There was effacement of foot processes overlying areas with amyloid deposition (Fig. 1c). A diagnosis of AA type amyloidosis was made.

### Clinical follow-up

Perindopril 2 mg once daily was initiated to lower proteinuria, but there was concern that it was exacerbating her anemia. Perindopril was discontinued when her hemoglobin had fallen to 47 g/L and a new anti-U antibody was detected, making red cell transfusion challenging. She was subsequently admitted to hospital with acute chest syndrome and mild pulmonary hypertension was noted on two-dimensional echocardiogram. Hydroxyurea was later initiated when her hemoglobin level was 61 g/L and discontinued 2 months later when her hemoglobin had fallen to 43 g/L, due to concern that hydroxyurea may have worsened her anemia. At the last available follow-up, 1 year after initial nephrology presentation, her Scr is 1.47 mg/dL (estimated GFR 51 mL/min/1.73m^2^), urine ACR 7965 mg/g and hemoglobin 66 g/L. Resting blood pressure is 129/75 mmHg. She does not have clinical features of systemic lupus erythematosus (SLE). Our patient has progressive chronic kidney disease due to AA amyloidosis. Her hematologist plans to reintroduce hydroxyurea at a lower starting dose.

## Discussion and conclusions

To our knowledge, this is the fifth report of AA amyloidosis among patients with homozygous sickle cell disease [2–5] and the first report with concomitant antibodies to double stranded DNA. AA amyloid protein, derived from serum amyloid A, is associated with chronic or recurring inflammation, such as found with chronic infection and rheumatic diseases. Recurrent vaso-occlusion with hypoxia and subsequent ischemia-reperfusion injury after resolution of vascular stasis, may lead to ongoing kidney inflammation and kidney injury in sickle cell disease [1]. It has been hypothesized that recurrent vaso-occlusive pain crises may lead to chronic inflammation, thereby leading to increase serum amyloid AA levels and ultimately leading to amyloid fibril formation [2, 5] but this is unclear. Furthermore, AA amyloidosis is associated with increased mortality, particularly if chronic kidney disease progresses to end-stage kidney disease [6].

Proteinuria in sickle cell disease increases with age [1] and is associated with hemolysis [7], as indicated by a lower hemoglobin level, higher LDH and higher reticulocyte count. Proteinuria is also associated with reduced GFR, as well as prior vaso-occlusive crisis, acute chest syndrome and pulmonary hypertension, all present in our patient [1]. Proteinuria was present in our patient 5 years prior to nephrology presentation but urine ACR values were not available again until 2 months prior to nephrology presentation, which prompted the nephrology referral. During the past 6 years, she has experienced multiple vaso-occlusive pain crises and acute chest syndrome and her estimated GFR has declined from 140 to 89 to 51 mL/min/1.73m^2^ over 6 years. She may have had glomerular hyperfiltration when her estimated GFR was 140 mL/min/1.73m^2^ and has now progressed to proteinuric chronic kidney disease due to AA amyloidosis. Although serum creatinine is an imperfect marker of glomerular filtration rate among patients with sickle cell disease because of increased tubular creatinine secretion [7], which is used in the CKD-EPI equation to calculate estimated GFR, this decline in estimated GFR is concerning. Preservation of kidney function may only be achieved by preventing further amyloid deposition into the kidneys. In AA amyloidosis, this requires control of the underlying disease, which in this case is sickle cell disease. Therapy that limits vaso-occlusive pain crises may limit further deposition of amyloid into the kidneys. Hydroxyurea, which induces fetal hemoglobin, is associated with decreased incidence of vaso-occlusive pain crises, number of red cell transfusions and proteinuria [8]. Angiotensin converting enzyme inhibitors are known to decrease proteinuria among patients with sickle cell disease and biopsy-proven glomerular enlargement and focal segmental glomerulosclerosis [9]. It is unknown whether ACE inhibition and hydroxyurea can reduce proteinuria or prevent worsening proteinuria in sickle cell disease with AA amyloidosis. Although initially unsuccessful, reintroduction of hydroxyurea at a lower starting dose may limit further vaso-occlusive crises and perhaps limit further AA amyloid deposition into the kidneys and progression of chronic kidney disease. Both hematology and nephrology teams felt that angiotensin converting enzyme inhibitor use was an unlikely contributor to our patient’s worsening anemia so reassessment for perindopril use is planned. Further investigations will be warranted if her anemia worsens again since hematology did not believe that erythropoietin would be of benefit.

There was an additional rare discovery of antibodies to double stranded DNA without other features of SLE. Anti-nuclear antibody positivity has been described among patients in 10–19% of patients with sickle cell disease compared with 0–2% of age- and sex-matched controls [10–13]. However, only 0–3% of these patients had antibodies to double stranded DNA. Although the presence of antibodies to double stranded DNA is only 73% specific for a diagnosis of SLE [14], it may portend a future risk of developing SLE [15]. Therefore, careful follow-up of our patient for the development of SLE is also required.

In summary, AA amyloidosis can occur in sickle cell disease. This case report highlights several teaching points. This case emphasizes the importance of increased awareness among nephrologists for AA amyloidosis as a cause of proteinuria in sickle cell disease. Nephrotic-range proteinuria is uncommon in sickle cell disease and kidney biopsy is necessary for diagnosis. Sickle cell disease can cause chronic kidney disease with proteinuria, reduced glomerular filtration rate and is associated with increased mortality [1]. AA amyloidosis in sickle cell disease may be caused by frequent vaso-occlusive crises so their prevention is important. Although unstudied in sickle cell disease with AA amyloidosis per se, ACE inhibition and hydroxyurea decrease proteinuria so they may limit progression of chronic kidney disease [8, 9]. Hydroxyurea decreases frequency of vaso-occlusive pain crises so it might be helpful in limiting progression of renal AA amyloidosis. However, further studies are needed to determine optimal treatment strategies for AA amyloidosis in sickle cell disease.

## Abbreviations

ACR: Albumin to creatinine ratio
CKD-EPI: Chronic Kidney Disease Epidemiology Collaboration
GFR: Glomerular Filtration Rate
SCD: Sickle cell disease
SLE: Systemic lupus erythematosus
